# Deconstructing Commercial Wearable Technology: Contributions toward Accurate and Free-Living Monitoring of Sleep

**DOI:** 10.3390/s21155071

**Published:** 2021-07-27

**Authors:** Lauren E. Rentz, Hana K. Ulman, Scott M. Galster

**Affiliations:** Human Performance Innovation Center, Rockefeller Neuroscience Institute, West Virginia University, Morgantown, WV 26505, USA; Lauren.Rentz@hsc.wvu.edu (L.E.R.); HKUlman@hsc.wvu.edu (H.K.U.)

**Keywords:** sleep, wearable device, wearable sensors, physiological monitoring, accuracy, consumer product, activity tracker, smartwatch

## Abstract

Despite prolific demands and sales, commercial sleep assessment is primarily limited by the inability to “measure” sleep itself; rather, secondary physiological signals are captured, combined, and subsequently classified as sleep or a specific sleep state. Using markedly different approaches compared with gold-standard polysomnography, wearable companies purporting to measure sleep have rapidly developed during recent decades. These devices are advertised to monitor sleep via sensors such as accelerometers, electrocardiography, photoplethysmography, and temperature, alone or in combination, to estimate sleep stage based upon physiological patterns. However, without regulatory oversight, this market has historically manufactured products of poor accuracy, and rarely with third-party validation. Specifically, these devices vary in their capacities to capture a signal of interest, process the signal, perform physiological calculations, and ultimately classify a state (sleep vs. wake) or sleep stage during a given time domain. Device performance depends largely on success in all the aforementioned requirements. Thus, this review provides context surrounding the complex hardware and software developed by wearable device companies in their attempts to estimate sleep-related phenomena, and outlines considerations and contributing factors for overall device success.

## 1. Introduction

Despite spending nearly a third of our lives in a restful state, the vast majority of individuals have limited knowledge surrounding sleep. Although why we sleep is still a question for many scientists, there is an abundance of research demonstrating the dangers associated with a lack of sleep, whether that be quality or quantity. Having implications regarding nearly all aspects of human performance and being deemed as a contributing factor for the development of many diseases, quantifying trends surrounding sleep are invaluable for personalized health and wellness monitoring.

The sleep medicine field has long sought a solution for the significant gap between the exceedingly limited, but accurate, polysomnographic methods and the ability to easily monitor free-living and longitudinal sleep patterns. The advent and proliferation of commercially available sleep technologies has acquiesced an alternative to measure sleep with increased accessibility and fewer limitations. These non-prescription devices that claim to monitor, track, and report sleep related metrics are comprised of wearables, nearables, and phone-based applications [1]. Sleep wearables offer a user-friendly, cost-effective solution to unobtrusively monitor sleep with enhanced comfort and convenience as compared to traditional sleep monitoring techniques [2]. These wearable devices have become increasingly popular in recent years due to the ease of data collection that they intend to offer.

The ease in usability offered by these devices substantially comes at the cost of accuracy; when considering the meticulous and thorough nature of polysomnography (PSG), it is impractical to suggest that a device costing less than a few hundred dollars could be a comparable alternative. Device consumers of all interested markets often fail to understand the intricacy and complexity of capturing sleep. Therefore, it is critical that end-users from all backgrounds understand the inherent capability of a device and its capacity to measure the intended metric(s) of interest.

The present review aims to enhance awareness and knowledge surrounding the utility of commercially available wearable devices to accurately estimate sleep related phenomena. Beginning with the physiology that is characteristic of the various states of consciousness, traditional methods of behavioral measures are delineated, along with the current methods deployed via sensors that are adopted by commercial wearables. In considering the applications of behavior captured by accelerometry, electrocardiography, and photoplethysmography, it is vital to understand that many of the signals captured are secondary in nature, and are assumed to correlate with specific behavioral (sleep) outcomes. This review highlights how secondary signals are used to estimate sleep related phenomena and how the combination of various signals contributes to the prediction of differing sleep states, clarifying the common sources of error and the variables that contribute to variations in accuracy across commercial products. Different sensor combinations, including their individual specifications, namely, plethysmography, in combination with signal acquisition and algorithmic functionality, help support the variation in efficacy that is reported by third-party validations. Finally, the applications and considerations of commercial wearables are evaluated for the use of sleep wearables by the consumer, researcher, and medical professional based upon the convergence of aims from the end-user and the technology, alike.

## 2. Sleep as a Physiologic Measure

Across states of alertness, the body functions with varying physiological patterns; namely, the brain operates at different frequencies, the tone of skeletal muscle varies, and the cardiovascular system executes at differing speeds and forces, all of which are dependent on the current state of an individual and the corresponding physiological function that the body aims to achieve. The same goes for sleep stages; as an individual transitions from one sleep stage to another, the physiological signals elicited by the body fluctuate. The status of these physiological measures provide insight regarding the state of an individual and are often used for sleep staging determinations.

During wakefulness, the body omits a compilation of physiological patterns that collectively and distinctly suggest if an individual is awake. The body acts with a relatively high degree of movement and muscle tone to support the execution of different tasks. Similarly, the eyes produce a high degree of movement, occurring as a general function of an active visual system. The brain functions predominantly at a frequency of around 16–25 Hz, producing mostly rhythmic beta waves with the presence of some alpha waves [3]. This pattern of electrical activity in the brain is common for minimal attentiveness.

As the waking body begins to tire and prepare for a restful state, these signals slightly shift; brain activity slows, becoming predominantly rhythmic alpha waves, to reflect the relaxed state accentuated by the closing of the eyes [4]. Concurrently, as the individual gets into bed, movement and muscle tone become slightly reduced as the body prepares to enter sleep. Also consistent with getting into bed is a slight reduction in blood pressure (BP), which results from the naturally occurring orthostatic response to laying horizontally [5], as well as the drop associated with sleep onset [6].

**Figure 1:**
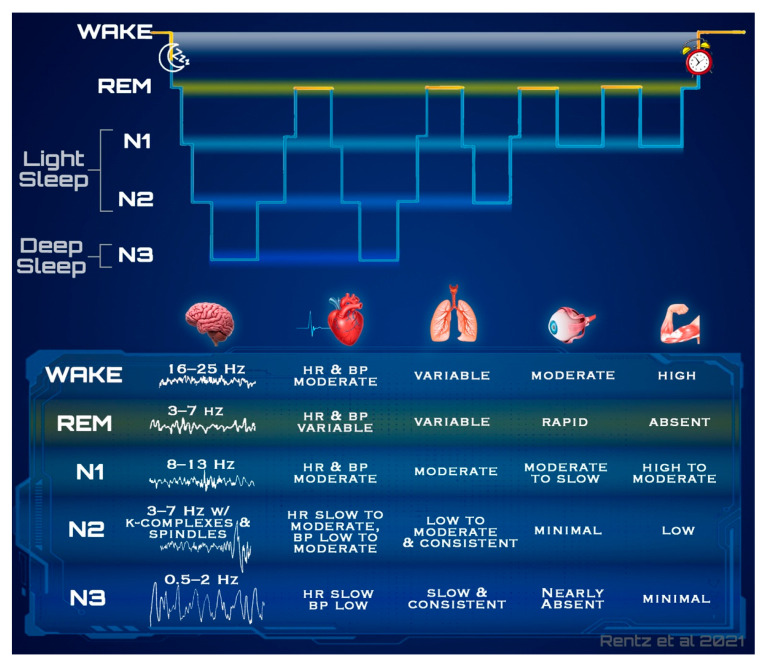
Systematic occurrences during wake and sleep. The figure demonstrates the minor physiological differences between wake and each of the sleep stages. Normal structural progression, displayed in the hypnogram at the top of the figure provides context surrounding the frequent shifts between states, and the subsequent reliance for accurately capturing physiological trends. Prominent physiological trends often characteristic of these individual states, which are each traditionally measured via separate methods, are comparable including brain activity, cardiac patterns (heart rate and blood pressure), respirations, eye movement, and muscle tone. REM physiology depicted is most representative of phasic REM. (BP, blood pressure; HR, heart rate; Hz, hertz; N1, non-rapid eye movement stage 1; N2, non-rapid eye movement stage 2; N3, non-rapid eye movement stage 3; REM, rapid eye movement).

Similar to wake, each stage of sleep has a unique combination of these physiological measures. Figure 1 outlines in more detail each of the precise physiological signal trends emitted by the body at different states, which each vary in difficulty of measurability. 

A hypnogram, as seen in Figure 1, is the archetypical depiction of an individuals’ sleep stages throughout the night. A typical sleep cycle progresses through NREM (non-rapid eye movement) sleep before entering a period of REM (rapid eye movement), which is often followed by a brief awakening. Sleep cycles ordinarily last 90 min on average and are repeated in a cyclic nature throughout the night, changing in structure as the night progresses [4,7]. Light sleep, which is a summative term used to collectively refer to both the first (N1) and second (N2) stages of NREM, is transitory in nature in that it often interpolates the transition between wake, N3, and REM. Light sleep has the lowest sensory threshold in which the brain utilizes this stage to perceive the highest aggregate of sensory stimuli from the environment whilst sleeping. Contrarily, deep sleep collectively refers to the stage three and previously named stage four of NREM sleep. Deep sleep occurs in greater durations in sleep cycles during the first half of the night, with only brief REM sleep durations; this balance progressively shifts throughout the night, gradually producing sleep cycles with shorter deep sleep durations and longer bouts of REM [4].

It is typical for the body to enter stage one of NREM sleep (N1) following sleep onset. While this is not always the case, in most healthy adults this lightest stage will commence the restful state. Many of the physiologic signals elicited are only minimally different to those of wake; muscle tone, heart rate (HR), blood pressure, and respiration rate (RR) present a slight reduction during N1 as compared to wake, and tend to stabilize and become more consistent [3,4]. Brain activity additionally exhibits a decline in frequency, containing fewer rhythmic alpha waves with a frequency of 8–13 Hz, shifting toward a greater composition of theta waves occurring around 3–7 Hz, which indicates a transition to an even slower state [7,8]. Though subtle, the most distinct physiological change occurring with sleep onset, is a gradual reduction in core body temperature of about 0.5 degrees Celsius, which reflects the decrease in temperature regulation [9].

Stage two of NREM sleep (N2), which commonly follows sleep onset and N1, presents only minute distinctions from lighter states via the physiological patterns elicited. Trends in muscle tone and RR are very similar to that of N1; however, spikes in HR and BP occur very briefly in response to the occurrence of eccentric brain activity that is predominantly unique to N2 [4]. Low amplitude mixed frequency brain waves continue, similar to those of N1, but additionally present with sleep spindles and K-complexes, which are instantaneous bursts of high and low activity respectively, lasting less than one second in duration [4,7]. In addition to the unique brain activity, these sleep spindles and K complexes are subsequently followed by abrupt increases in HR and BP. The presence of these unique periods with otherwise downward trending HRs, are a few of the physiological trends that suggest the occurrence of N2.

Stage three of NREM sleep (N3), which includes the stage four NREM determination previously used, is termed “deep sleep.” The highest sensory threshold occurs during this stage, which is reflected by the low degree of muscle tone and movement, and the occurrence of the slowest brain waves [3,8]. The brain exhibits very slow activity with high voltage during N3, displaying a large presence of delta waves with a frequency around 0.5–2 Hz. Further, HR, BP, and RR remain consistent, but are reduced from that of wake. Muscle tone, however, is greatly reduced and eye activity presents the fewest movements in this stage [3,7,8]. Following N3, it is common to re-enter N2 and eventually N1 before beginning REM; however, there are many situations in which behavior deviates from this pattern.

Substantially the most variable stage, REM or paradoxical sleep, is most strongly characterized by its suppression of muscle tone throughout the body coupled with a significant number of rapid movements of the eyes. Though these physiological trends are indicative of phasic REM periods, the microstructure of REM sleep also involves periods of tonic REM, which elicits notably different physiological signals [10]. Brain activity during REM is predominantly mixed frequency waves of low voltage, primarily manifesting as theta waves in the 3–8 Hz range in much of the brain, aside from faster beta waves of 15–35 Hz in regions of the frontal lobe [11]. Because phasic and tonic components of REM sleep involve significant autonomic activation of the sympathetic and parasympathetic systems, respectfully, cardiac and respiratory patterns are of high variation. During tonic REM, HR is typically low and similar to that of NREM, whereas phasic REM results in HR increases with much variation, which has been theorized to coincide with dreaming activity [12]. Of note, peripheral BP tends to decrease during both tonic and phasic REM by result of the increased shunting of blood flow to the brain [13].

## 3. Traditional Methods of Sleep Assessment

Human sleep experiments are traditionally conducted in laboratory-based settings using polysomnography (PSG), which continuously monitors cardiorespiratory and neurophysiological changes in the body to characterize sleep related phenomena [14]. PSG is a multi-parametric evaluation that utilizes electroencephalography (EEG), electromyography (EMG), and electrooculography (EOG) measures to assess the electrical activity of the brain, degree of muscle tone (generally of the chin and limbs), and eye movement, respectively [15]. Concurrently, cardiac activity is measured via electrocardiographic (ECG) leads placed on the chest, blood oxygen levels through pulse oximetry, and respiration patterns. This industry accepted, “gold standard” group of physiological measures plays a crucial role in obtaining the most accurate information required for differentiation of wakefulness from sleep, sleep stage classification, and the diagnosis of sleep-related disorders [14].

Following collection, data captured from each of the individual signals is individually appraised in 30 s segments, termed epochs, that are then manually categorized by technicians according to standardized criteria. There are currently two different criteria for stage classification; standards from the American Academy of Sleep Medicine (AASM), which is more widely used, and criteria from Rechtscaffen and Kales. AASM scoring classifies epochs as either wake, N1, N2, N3, or REM, where N signifies the three stages of non-rapid eye movement sleep (NREM) and REM represents rapid eye movement sleep (REM) [16]. Criteria from Rechtschaffen and Kales classifies epochs as either wake, S1, S2, S3, S4, or REM, where S signifies stages one through four of NREM sleep [17]. Due to the nature of manual classification, there are reportedly large variations in interrater reliability (IRR), with interrater agreements sometimes as low as 70% [18,19,20,21]. Measures to mitigate error often include repeated analysis of the data epochs by different technicians in which results are then aggregated.

Though most accurate, PSG is an arduous and expensive endeavor and must be conducted in a controlled, laboratory environment in the presence of skilled technicians. In an attempt to simplify the complexity of sleep monitoring via PSG, actigraphy has become a popular alternative, providing an indirect measure of sleep by assessing movement through a wearable, wrist-based device. Unlike most PSG assessments, actigraphy can measure sleep in the comfort of an individual’s own home, reducing the “first night affect,” and providing the opportunity to assess normative sleep patterns. Although considered a reliable method for sleep research, actigraphy is limited to only wake vs. sleep classifications [2,22]. Further, actigraphy is associated with high error rates and commonly overestimates sleep in many individuals [2,23]. This method is known to substantiate artifacts related to breathing movements, postural blocking of arm movement, low activity tasks, and external movement [22,24].

Well-documented sleep logs can help to ascertain artifact from signal; users are often asked to keep a well-documented sleep log to ensure sufficient removal of the unwanted data. This task is not only a burden for the researcher and consumer, but it also presents a new opportunity for error [22]. Subjective reports of sleep lack significant insight on physiology, and of the measures that individuals are able to report on, research still often demonstrates poor degrees of accuracy [25,26]. The technology to accurately quantify sleep undoubtedly exists, but the associated limitations largely affect the accessibility and applications for evaluation of sleep.

Both PSG and actigraphy are used in research and clinical settings; however, the applicability of these methods on a larger scale is limited by the level of expertise and cost associated with operating such specialized equipment and software used to perform sleep analysis. Through the advent and proliferation of sensor capabilities, advanced computing power of mobile devices, analysis techniques, and storage capacity, bimodal sleep technologies are emerging on the market as an attempted solution to bridge the gap in limitations and allow for sleep analysis in a free-living environment.

## 4. Device Capabilities and Methods of Measure

Wearable devices that purport to assess sleep often utilize a combinatorial approach; this technique incorporates one or more embedded sensors to obtain various measures that estimate physiological signals, which are further used in attempts to classify sleep-related physiology [27]. Common sensor types that are often embedded in emerging wearables include accelerometers, electrocardiographs, gyroscopes, photoplethysmographs (PPG), and thermal sensors. The aforementioned sensors each provide valuable insight regarding physiological parameters that vary in their capacity to estimate sleep related phenomena.

Table 1 contains common commercially available sleep wearables and include the embedded mechanisms they use to collect these parameters. These devices often incorporate multiple sensor types, that when combined, have the opportunity for improved accuracy of sleep classification. It is important to note that though many of these devices utilize similar types of technology, the sensors themselves are likely to be manufactured with varying specifications and capacities to measure the output of interest. An in-depth evaluation of each of the sensors are described in the following sections.

### 4.1. Accelerometers and Gyroscopes

Accelerometers measure movement in terms of changes in velocity, providing input on the frequency, duration, and intensity of motion [28,29]; they are the most commonly used method in wearable device motion-sensing and are the primary sensor used in actigraphy devices [27,29]. Gyroscopes are devices that can effectively measure the changes in angular velocity, or orientation, to determine the direction at which the object or person is turning [30]. Accelerometers and gyroscopes are often used in unison as the outputs of each device can be used to compliment the other in understanding changes related to movement.

The mechanical innerworkings of accelerometers and gyroscopes are beyond the scope of this paper; however, it is important to note that the number of axes may vary among devices. For instance, both accelerometers and gyroscopes can have a one-, two-, or three-dimensional axis (x,y,z) that each pertain to movements of different directions [30]. The distinction among the number of axes plays an important role in distinguishing linear or angular acceleration from orientation. For example, a 3-axis accelerometer can sense the orientation of a stationary object relative to the Earth’s surface, but it is unable to distinguish the acceleration of an object from the acceleration of gravity [30,31]. Meanwhile, gyroscopes use the Earth’s gravitational pull in order to calculate the orientation or angular position of an object in space, or the tilt and turn of an object; however, unlike accelerometers, gyroscopes are unable to measure the direction of acceleration [30]. Therefore, the most promising results implement a combinatorial approach of embedding both an accelerometer and gyroscope into a wearable device [30,31].

Because of the relatively simplistic nature of accelerometers, these devices are susceptible to error and can incorrectly classify low activity tasks [24]. With specific regards to measuring sleep, accelerometers can be affected by other movements made in the bed, such as a partner rolling over or a pet jumping into bed. Additionally, accelerometers are subject to inaccurate recordings in patients with neuromuscular impairments that may cause tremors in the hand or body [32]. Nonetheless, when combined with other physiological sensors, accelerometers can provide crucial context when classifying the activity of an individual, especially when in relation to sleep and wakefulness.

### 4.2. Electrocardiography

Multi-lead electrocardiograph (ECG) outputs have been deemed the prominent secondary signal to assess sleep staging, secondary to EEG [15]. ECG measures the electrical activity of the heart using electrodes that are placed on the surface of the skin [33]. ECG devices use the conductance generated by the cardiovascular system to produce the PQRST waveform characteristic of depolarization and repolarization of the various heart chambers; from this wave, beat to beat heart rate and heart rate variability (HRV) related metrics can be derived using the inter-beat-interval (IBI) [34,35]. IBI data represent the time interval (in milliseconds) between two successive R peaks.

ECG devices can vary in the number of leads that are attached to the device. Conventionally, assessment of cardiac functionality is obtained in a clinical setting using 12+ lead ECG [36], where electrodes are placed in close proximity to the heart to ensure that the signal to noise ratio remains minimal and constant [37]. The sampling frequencies of multi-lead, clinical grade ECGs typically range from 250 to 500 Hz or higher [23,38,39], though wearable devices often have much lower sampling rates to preserve battery life. Multi-lead ECG devices, however, are not practical for the consumer population due to the critical relative placement of electrodes and the technical expertise needed to interpret the generated signal; thus, single lead ECG-based sensors have been embedded into wearable devices, requiring minimal area of skin contact and reducing the opportunity for user error. Despite the ease and utility of a single channel lead, there are known implications of recording from only one source. Notably, single channel ECG are known to underestimate the durations of the waveform complex [40].

Ultimately, the quality of the ECG signal is influenced by a multitude of factors, whether it be the sampling frequency, number of leads, or how the signal is interpreted. Following signal acquisition, the ECG signal must undergo a series of signal processing steps that include filtering, transformation, waveform recognition, feature extraction, and diagnostic classification [40]. However, the aforementioned method to effectively characterize subsequent peaks from one another varies from one source to the next. Signal processing of an ECG signal is often necessary as the devices are susceptible to artifact that presents as baseline wander due to chest movement induced by respiration, issues with electrode impedance, technical failures, or ectopic beats (e.g., arrhythmias) that result in missing or double beats, further troubled by low sampling frequencies [2]. Various noise cleaning and artifact detection algorithms have been proposed that utilize filtering techniques to remove baseline wander, in combination with adaptative and moving thresholds to effectively eliminate unwanted artifacts. Often, these noise-reduction algorithms are proprietary in nature, leaving it unbeknownst to the end-user what is going on in the background.

### 4.3. Plethysmography

Photo, meaning “light”, and plethysmograph, meaning “measurement of changes in volume,” directly translates to mean *the measure of volumetric changes through use of light*. Photoplethysmography (PPG) is a common technology utilized in wearable devices that aims to detect volumetric changes in distal arterial blood flow [41] by quantifying the amount of light transmitted or reflected back to the photodetector [42,43]. The output of the device is a pulsatile waveform that is sinusoidal (smoothened, periodical oscillations) in nature, opposed to the distinctive R peaks found in an ECG.

Analysis of this waveform occurs using two distinct comparisons of the reflected signal over time: alternating current (AC) and direct current (DC). The AC primarily measures the highs and lows of vessel volume, reflective of alternating between systolic and diastolic pressures respectively [44]. The DC focuses on the variation in the strength of reflected light between repeated highs and lows; which can vary from influence of other physiological occurrences, such as respirations. PPG sensors vary in their form, which generally coincide with their intended use. Contact PPG in which the sensor has direct contact with the skin is most often used in wearable devices but vary in the components and arrangement of the sensor.

#### 4.3.1. Components of Sensors

PPG sensors can contain different light sources that vary in their wavelength and subsequent capacity to penetrate the skin. Namely, wearable devices generally either utilize a green light emitting diode (LED) or a combination of red LED and infrared, ranging around 520–560 nm and 800+ nm, respectively. The differences in wavelengths have varying implications that could affect signal quality and accurate reporting. It is generally postulated that the higher the wavelength of light, the deeper it is able to infiltrate the tissue. For instance, higher wavelengths used in red or infrared light sources are less susceptible to variations in error due to skin color [45,46]. The shorter the wavelength, the more readily the light is absorbed by melanin; this phenomenon is a consequence of the optical water window. Namely, tissue is primarily composed of water, which more readily absorbs light in the ultraviolet or longer infrared spectrum [42]. Albeit, there exists a small window where red and near infrared wavelengths are able to pass through tissue with greater ease. Thus, green LED light sources present greater susceptibilities for error from variations in skin color, as the wavelength is more readily absorbed by melanin pigments in the skin, resulting in high variations in the amount of light able to return to the sensor, independent of blood vessel properties [42,45,46].

Despite the shortcomings of green LED in individuals with darker complexions, green light is more adept at measuring heart rate by consequence of the wavelength [31,45]. Because the green LED does not penetrate the skin as deeply, its signal is less position dependent, primarily targeting superficial vessels [44]. Contrarily, red and infrared LEDs surpass the capabilities of the green LED when measuring HRV parameters [47]; because the light is able to penetrate deeper into the skin, it is able to interact with larger arterial vessels that are positioned deeper in the tissue, which demonstrate greater volumetric fluctuations with each heartbeat [44,48]. Furthermore, the differences in coloration have a known impact in the absence or presence of motion, with red and infrared outperforming green wavelengths during rest, such as during sleep, but often green outperforms the higher wavelengths during periods of higher activity or motion.

It should be noted that sampling frequency has known implications on signal quality and overall device performance, which is the case for PPG sensors as well as the other sensors discussed herein. Both green and red LED/infrared light sources perform with greater accuracy at a higher sampling frequency; higher sampling frequencies are able to more effectively detect changes in the absorbed or reflected wavelengths as the blood volume and pressure changes over time [31,49].

Signal quality influenced by ambient light is another notable factor, as all PPG sensors use some form of a light source and photodetector. Like ECG based devices, contact PPG sensors should always lay flush against the skin, with sufficient pressure to capture readings, without applying too much pressure that could restrict blood flow in underlying tissues [50]. Thus, if a device is attached loosely to the skin, the amount of light transmitted or reflected back to the photodetector may not be truly indicative of the signal at hand.

#### 4.3.2. Arrangement of Sensors

**Figure 2:**
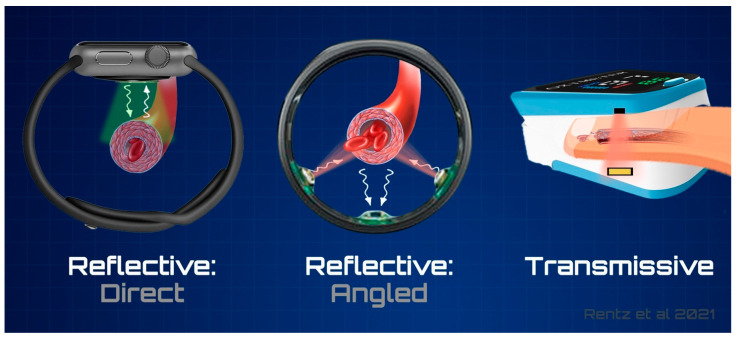
Arrangements of Photoplethysmography (PPG) Sensor Components. The two primary arrangements of PPG sensors, Reflective and Transmissive, as they pertain to the position of the light source and sensor in relation to target blood vessels. Reflective arrangements are further segregated based upon the relative positioning of the light source and sensor as either direct or angled.

There are two basic arrangements of PPG sensor components that comprise the wearable PPG device market; transmissive and reflective [44]. As shown in Figure 2, transmissive sensors use a light emitting source that is detected by a photodetector located on the opposite side of a tissue. This type of sensor is limited to measurement of certain anatomical locations, such as at the fingertips or earlobes, which are thin enough for the signal to penetrate. In contrast, reflective sensors detect the amount of light that is reflected or back scattered from the tissue to the adjacent photodetector. Unlike transmissive sensors, reflective configurations have less constraints on the placement of the sensor; resultingly, reflective PPG offers a more convenient configuration for end-users as these devices can be easily placed around the wrist, or on anatomical locations with thicker tissues. As a result of its reflective properties and its more superficial targets, green LED is more widely utilized in a reflective arrangement, are rarely seen in transmissive PPG; however, infrared and red LEDs are often utilized in both arrangements. Device manufacturers have also started designing devices with numerous light sources of varying wavelengths, as seen in the Polar Grit X, understanding that they each demonstrate benefits and limitations under different conditions.

Many commercial devices utilizing PPG sensors are arranged in a manner that requires the reflecting signal to return to almost the exact location that it was emitted from; this direct reflection places a high dependency on proper sensor placement to ensure that it is positioned above the target blood vessel. Primarily seen in wrist-based devices, this arrangement presents the opportunity for error resulting from slight variations in anatomy. Risk of sensor misplacement can be mitigated through slight alterations in component arrangement; by separating the light source from the photodetector and including multiple light sources, the likelihood of interacting with the target vessel increases as a result of reduced positional dependency. This angled reflective arrangement is demonstrated in Figure 2. Angled arrangements, however, are often avoided due to the required customizability of the product to gross anatomy (i.e., wrist or finger circumference); for more universal applications, wrist-based devices are typically designed to fit all sensor components on the watch back, allowing for easier exchange of watchbands based on customer preference.

Due to the positional dependency of PPG devices (most commonly placed at the most distal parts of the body such as the wrist, ankle, or finger), the signal is highly susceptible to motion artifacts, which can greatly affect data accuracy [47,51,52]. Namely, motion artifact disrupts the quality of the signal from blood vessel volumetric trends, especially during bouts of high intensity activities. This particular issue should have little impact on sleep staging classifications, unless the device was designed to cater to converging aims such as exercise monitoring, which will be discussed in more detail in later sections. To obtain a cleaner, more representative signal, noise cleaning algorithms are often incorporated into the overall system architecture.

### 4.4. Temperature Sensors

Measures of body temperature can be estimated from skin temperature using a variety of sensors that may include infrared thermopiles, thermistors, thermoelectric effects, or measure via optical methods [53]. These sensors vary in their capacity to estimate body temperature, as some methods are more vulnerable to variations in ambient temperature. The direction at which a sensor faces and the degree of contact can make a sensor more susceptible to environmental influences.

Temperature sensors vary largely in the data in which they collect. In general, these sensors provide indirect measures of temperature based upon characteristics of an adjacent tissue. Sensors containing thermistors, which can include negative or positive temperature coefficients (NTC and PTC, respectively), measure a value of material resistance for conductivity [54]. Thermoelectric sensors utilize a measure of voltage that is carried through a material or tissue, which will vary based upon the temperature of the tissue. Other optical and infrared thermopile sensors measure the interaction between infrared light and the tissue of interest, basing temperature estimations from different response properties.

## 5. Application of Sensors for Sleep Estimation

Arguably the most significant limitation of “measuring sleep” is simply the inability to measure the key variable (sleep); instead, sleep can only be estimated through quantification of the physiological elicitations that are characteristic of a given state. Sensors of wearable devices are able to capture some of the physiological trends that occur during sleep, either directly from primary signals or indirectly through interpretation of collected signals. Though some physiology is virtually impossible to measure through typical limb-mounted devices, sensors determining concurrent behavior of the muscular, nervous, cardiac, and respiratory systems are among common methods deployed in attempts to estimate sleep.

### 5.1. Sensor Utility in Capturing Physiology

A broad understanding of neuromotor activity can be achieved from data collected from accelerometers and gyroscopes. Movement is captured often in terms of acceleration (change in velocity over time) and can be used to quantify activity intensity. Postural dependencies (standing vs. lying down) can be determined by gyroscopes or the combination of accelerometer data with time to derive velocity and displacement. Collectively, these measured patterns in movement are suggested to coincide with muscle tone and motor stimulation. During sleep, human motion typically occurs at a rate of 1–25 Hz, which can then be expressed in activity counts [55]. It is thought that movement above a specific threshold is indicative of wake due to the high muscle tone and freedom of motor activity at this state. The main limitation with accelerometers and gyroscopes is the assumption that a “lack of movement” is sleep, thus implying that motionless wake intervals are likely to be incorrectly classified as sleep. This leads to overestimations in total sleep time and sleep efficiency, as well as underestimations in sleep onset latency (SOL) and wake time after sleep onset (WASO) [56].

**Figure 3:**
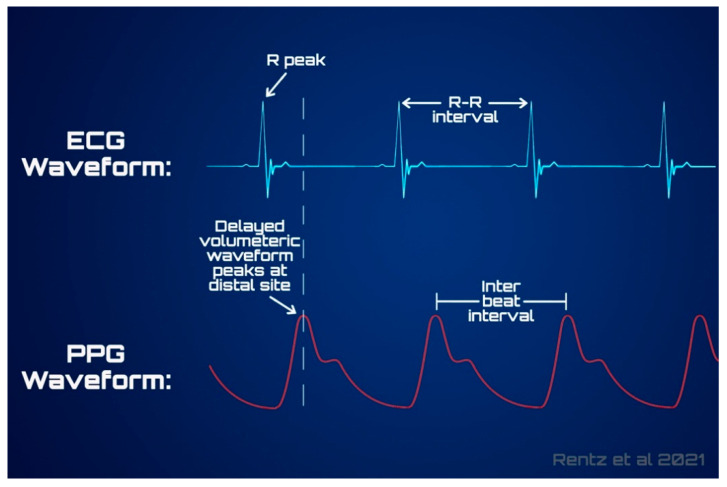
A comparison of cardiovascular signals in wearable devices. The electrocardiogram (ECG) waveform is shown relative to the volumetric waveform obtained via photoplethysmography (PPG). Of note, the volumetric changes that are demonstrated in the PPG waveform captured at a distal anatomical location travel slower than the electrical impulses that are captured by ECG, resulting in a slight delay of features, though highly proportional to that of ECG.

Cardiac behavior is often evaluated using one of two methods: directly through electrical waveforms from ECG, or indirectly through the pulse pressure waveform (PPW) from PPG. ECG waveforms are composed of PQRST waves that are indicative of electrical stimulation for the opening and closing of chambers and valves within the heart [34,35]. When collected using a single lead from a distal location, such as the wrist, the most notable depiction is of the R peaks, which can be seen in Figure 3. This maximum voltage value indicates stimulation for contraction of the heart’s ventricles and is the most distinct marker suggestive of a heartbeat [34]. General rate of R peak occurrence from the ECG signal provides a measure of HR. Similarly, relative variation in the time between consecutive R peaks for a given time frame contributes to calculation of HRV variables. This direct measure of conductivity waveform allows for considerable accuracy surrounding HRV; these variables can provide insight as to the balance between sympathetic and parasympathetic autonomic activity, offering a valuable set of measures when determining sleep trends [57,58,59].

The other method of observation surrounding activity of the cardiac system is through the PPW measured via PPG. Through utilization of sensor components, PPG measures the resultant strength of the reflected light and estimates the variations in blood volume from a vessel carrying blood away from the heart. These vessels expand as a result of the increased pressures from heart contraction, producing a waveform that is also depicted in Figure 3 as it compares to the output of ECG. The pulsatile AC component is used to measure pulse rate as a surrogate for HR and HRV related metrics, which are calculated from the peaks in the PPW, similar to the IBI calculation from ECG R-R intervals [44,60].

PPG waveforms can also provide insight on physiology and bio-signals beyond HR and HRV; in combination with the AC component, the DC component can be used to estimate patterns in RR and peripheral resistance [50,60], both of which can vary with sleep arousals. As respirations occur, the highs and lows of the reflected light signal strength vary, which becomes superimposed on the shape of the AC component. The more subtle fluctuations in signal strength can provide insight on the inflation and deflation of the lungs [44,60,61]. RR is particularly useful in discriminating between sleep stages, with more stable and regular RR amplitude and frequency patterns indicative of non-REM, particularly deep sleep.

Temperature sensors are not as widely utilized in classifying sleep due to their high susceptibility to be influenced by external and internal factors. Collectively, these sensors may aim to measure overall peripheral body temperature or skin temperature. While theorized to relate to core body temperature, known to decrease with the occurrence of sleep onset, trends in peripheral temperature are more indicative of neural thermoregulatory control via vasoconstriction and vasodilation of the blood vessels in the hands and feet [9,62]. Though core body temperatures tend to vary by less than 1 °C throughout the night, skin or peripheral temperature variations can span as drastically as 2–3 °C [62]. With this, there are converging theories surrounding the relationship between the temperature of the periphery, where these sensors are located, and core body temperature during sleep. Theories speculate as to whether the two are consistently reflective of the same trend or whether peripheral thermoregulation can act in a converging manner to contribute to core body temperature fluctuations [9,62,63]. Additionally, internal factors such as fever or menstrual cycles are known to cause variations in body temperature, deeming this a difficult variable for wearable devices to rely on. The added complexity surrounding temperature sensors relative to their insight in quantifying sleep often results in their exclusion from devices altogether; if included, these sensors are most commonly used as a variable contributing to the determination of sleep onset or sleep quality based on individualized normative values.

### 5.2. Device Claims vs. Methodologial Capabilities

As behavior modulates during sleep, all equipped sensors for a device will concurrently collect any physiological measures in which they are capable of quantifying. As Figure 1 demonstrated previously, there is much overlap in the signals capable of measurement via the sensors explained herein. Thus, it is the behavioral insight that is obtained from various perspectives that allow for state classification, or further sleep staging. Comparatively, wearable devices estimate only a portion of these physiological signals, making it a significant challenge to confidently classify a stage to each epoch.

Traditionally, wearable devices have struggled with identifying the precise timing for which an individual transitions from a relaxed, wakeful state into sleep [64]. Patterns for HR, HRV, and RR are of high similarity based upon relaxation and increased parasympathetic tone; however, previous research has focused specifically on physiology during this period, finding that quantifiable differences exist in HRV parameters immediately surrounding sleep onset based upon subtypes in sleep behavior [65]. Further, little movement often occurs during this transition, making determinations of sleep onset difficult, which has been well demonstrated across validation studies [64,66,67]. Though for years actigraphy has been deemed an acceptable method for differentiating sleep vs. wake, wakeful periods of high relaxation and no movement have been commonly known to be incorrectly classified as sleep [68]. Without EEG and EMG signals to aid in this determination, a combination of relative HR, HRV, and RR parameters must suffice for sleep onset classifications. In devices equipped with temperature sensors, the decrease in body temperature associated with sleep onset can provide valuable information to augment this designation.

Following the initial onset of sleep, fluctuations in movement, HR, HRV, and RR parameters can provide notable value for sleep stage differentiation, though with different methodology as compared to the feature identification and epoch classification of traditional PSG. EEG provides, unarguably, the greatest insight on a given sleep stage, including those of NREM; sleep spindles and K complexes, signature features of stage 2, as well as the characteristic slow waves of stage 3, are not identifiable through most commercial wearables utilizing the sensors explained herein. During sleep, these cortical oscillations are of high relation to autonomic tone at a given point, demonstrating strong relationships with the balance of sympathetic and parasympathetic activity [69]. Despite the abrupt increases in HR and BP that accompany spontaneous cortical activity such as sleep spindles, these patterns are not reported to be the focus of associated feature extraction in automated scoring; without EEG to affirm the occurrence of a sleep spindle, this altered waveform displayed in sensor signals could be caused by numerous factors. Rather, peaks in the pulsatory waveform are used for extensive HRV calculations, which are not traditionally calculated for PSG scoring [57,58,59,70,71,72,73]. Calculations such as low frequency (LF), very low frequency (VLF), and high frequency (HF) are often used to quantify time sensitive variations in pulsatile wave characteristics. Thus, these cardiac patterns will be reflected in composite HRV parameters, which have been found to aid in the differentiation of light from slow wave sleep [57]. These HRV parameters are suggested to be significant indicators of sympathetic activity (primarily LF and VLF) and vagal-nerve, or parasympathetic, activity (primarily HF) [74]. Similarly, respiratory patterns are derived from variations in waveform shape (i.e., the DC component of the PPW), and provide context on respiratory rate, breath cycle, and breath variability, which are also key indicators of autonomic balance [58,75,76]. The lack in EEG and EMG signals, traditionally vital contributors to NREM staging during PSG, demonstrate the requirement for a different approach to physiological sleep stage classification.

Of most difficulty, epoch classification as REM sleep using the physiological parameters captured via wearable devices is challenged by the scarcity of identifying features for the stage. Specifically, wearables lack sensors capable of providing insight regarding muscular atonia and rapid eye movements, which are signature attributes of REM sleep traditionally captured via EMG and EOG, respectively. Instead, the lack of muscle tone is tied to the lack of movement captured by accelerometers, though the limitations of this assumption are widely recognized in validation studies as being insufficient [68]. Wearable signals also lack demonstration of sawtooth EEG waves, further limiting context on this otherwise easily distinguishable stage when assessed via PSG. The only resulting physiological trends that can provide discernment on the existence of REM behavior are trends in HR, HRV, and RR that wearables often quantify via PPG. As discussed previously, these measures can demonstrate much variability during REM sleep, unlike other stages; notable spikes in HR and RR can occur just as frequently as mellow lows [58,70,71,77], suggesting these epochs of the often called “paradoxical sleep” can easily be confused with wake and light sleep, respectively [10]. Without other data types to ascertain these assumptions that are based primarily on cardiac and respiratory trends, confidently classifying a given epoch as REM is of high difficulty. 

## 6. System Architecture for Classifying Sleep

Sleep monitoring systems have assuredly progressed throughout the ages, due to the rapid growth of the Internet of things, which includes the development of more sophisticated sensors and software, and advancements in the storage of big data. Although the advancements in technology make automated sleep classification systems more achievable, the classification of sleep and its respective stages remains an arduous and challenging task. Namely, sleep is a culmination of complex physiological and psychophysiological variables that cannot be directly captured or quantified. As mentioned previously, sleep is “captured” using secondary signals that aim to provide some level of understanding surrounding an individual’s state of wakefulness. Capturing the physiological metric(s) of interest is only the first step in an overall architecture that intends to classify an instance as sleep vs. wake, or as a sleep stage.

Behavioral patterns associated with changes in sleep stage modulate specific physiologic patterns that can be detected by wearable sensors just as they can be via traditional monitoring methods utilized in PSG. An algorithm, or series of problem-solving operations can be used to determine how the patterns or combinations of patterns are related in their ability to classify a specific state or stage. The patterns can be recognized and manipulated by mathematical equations that find commonalities between the ingested bio-signals (e.g., increase in HR and acceleration could suggest a wakeful state) and subsequently apply them to a categorical classifier (e.g., sleep vs. wake, or wake vs. light vs. deep vs. REM). Studies have demonstrated that parameters associated with respiration, heart rate, and movement are most adept at sleep stage recognition [78]. Specifically, a recent study published by Altini and Kinnunen (2021) demonstrated that device accuracy for four stage detection increased from 57% to 79% when using accelerometer, temperature, HRV, and circadian features rather than just accelerometry alone [77]. Similarly, studies published by Fonseca et al. (2017) and Walch et al. (2019) also found sleep algorithm accuracy to increase following the addition of PPG data, as compared to accelerometer data alone [70,79].

Traditionally, sleep monitoring in the free-living environment was driven by actigraphy devices that used accelerometers to link changes in motion to differentiate sleep and wakefulness, with reported accuracies typically varying from 80% to 95% [80,81]. Currently, there are several validated and publicly available algorithms that provide a binary classification of sleep or wake (1 for sleep, 0 for wake), such as the Cole–Kripke or Sadeh algorithms [29,82,83]. The new generation of sleep monitoring utilizes a combinatorial approach of multiple sensor types to distinguish changes in sleep macrostructure, specifically sleep staging, via the collection of multiple bio-signals. Multiple measurements provide an added layer of redundancy that improves the quality of algorithm classification, as some signals may overlook a particular bit of information that may subsequently be captured by another. Additionally, some signals do not generalize well for some users. For instance, accelerometers largely overestimate sleep in those that do not move a lot, while underestimating sleep in restless sleepers or those sleeping with a partner or pets. This initially led to further alterations of movement thresholds for some algorithms, like separation into “sensitive” and “normal” modes as done previously by Fitbit [84]. The combination of multiple signal types undeniably requires more robust methods to effectively collate and manipulate signal types from various sensors of varying sampling frequencies, resolution, and quality.

Algorithm accuracy is largely driven by signal quality; therefore, noise cleaning algorithms aim to remove unwanted artifacts (associated with movement, technical failures, ectopic beats, etc.) that disrupt the overall signal quality. Following the acquisition of the signals of interest, signal processing schemes are often embedded within the device’s firmware, noted by the real-time display of metrics such as HR, on the user interface of the device. The types of techniques used to clean signals vary based on the type of sensor and signal that they aim to capture; further, these noise-cleaning algorithms vary alongside sensor specifications and sampling frequencies and are often proprietary.

Sleep classification algorithms utilize machine learning techniques to effectively “learn” from a given dataset and make informed decisions surrounding sleep or wake, or further into sleep staging. Typically, sleep algorithms utilize supervised techniques using pre-labeled PSG data collected in a clinical setting [2,24]. It should be noted that this is an onerous undertaking, as these algorithms require a large amount of training data to produce accurate results; otherwise, a small sample size translates to a lack of inter-individual generalizability and subsequent over-fitting of the model. Training models are fundamentally influenced by inter-technician PSG scoring and inconsistencies in noise cleaning algorithms between different manufacturing companies, often leading to diminished overall accuracy [2]. Prior to model execution, an extensive amount of pre-processing must be implemented to ensure proper synchronization and temporal alignment of PSG and wearable device data streams, with consideration for peripherally positioned sensors. This guarantees that there is no clock drift or variations in the start time between the two or more signals [70].

Automated systems are advantageous as they reduce inter- and intra-observer variability and decrease valuable human resource time; however, the computational cost associated with handling such large amounts of data can significantly limit the usability and applicability of such an algorithm in a “real-world” setting. Therefore, feature selection is performed to reduce the amount of data used to ultimately train the algorithm through the selection, reduction, and combination of certain variables (i.e., pertinent bio-signals). Features can include parameters such as HR, activity count, rotation magnitude, and heart-rate-variability measures or can be represented as a mathematical combination of such measures using transformations such as continuous and discrete Wavelet transformations, Short-time Fourier transform (STFT), spectrograms, Choi Williams distributions, ensemble empirical mode decompositions, or higher order statistics (HOS) [15,85]. Similar to the analysis of PSG, features are extracted across epochs, traditionally a 30 s time window, to mimic the technique utilized by human PSG scorers that look at the relationship between past and future epochs. Features then undergo normalization so that one parameter will not initially have more weight than another, and then fed into a classifier with associated labels (e.g., “wake”, “light”, “deep”, “REM”) [73]. Normalization methods include, but are not limited to, z-score normalization or spline smoothing.

Of the classification-based machine learning models used, K-nearest neighbors (KNN), Support Vector Machines (SVM), Random Forest, Artificial Neural Networks (ANN), Naïve Bayes (NB), Gradient Boosting Decision Trees (XGBoost), Gaussian Mixture Model (GMM), Hidden Markov Model (HMM), and Linear Discriminant Analysis (LDA) remain among the most popular [15,85,86]. When choosing a machine learning model, it is important to consider model characteristics and tendencies, in addition to the available time, hardware, and software resources needed to implement any given model [87]. For instance, LDA models are known to be robust to variations in datasets [73], which is important when considering the day-to-day discrepancies in sleep patterns and the inherent disparity across participants. NB, random forests, and SVM algorithms are simplistic and computationally efficient making them adept at binary classification problems, which has applications in classifying sleep vs. wake [88]. By the same token, larger ANNs are robust at multi-class labeling but are susceptible to overfitting (i.e., conforming too tightly to the training dataset, resulting in less generalizability when exposed to new datasets) [87]. Alternatively, sleep algorithms can utilize unsupervised techniques that do not require the need for an expert technician or labeled data; however, it is unlikely that unsupervised training algorithms are able to achieve better accuracy than a supervised model [24] as the results of PSG scoring may be challenging for the computer. For instance, clustering algorithms attempt to find ‘clusters’ or ‘groupings’ of similar datapoints and assign them to a specific category. In the case of sleep staging classification, the physiologic patterns tend to overlap, making it immensely difficult for a computer to the subtle differences in sleep stages from one another without a prelabeled reference point.

It is paramount to understand that comparing algorithm performance across approaches is not a simple task. A model’s accuracy can be influenced by a multitude of factors including but not limited to, number of sleep stages considered, pertinent sensor types and number of channels, the type and quantity of extracted features, the chosen classification and validation methodology, and the performance evaluation metrics [89]. However, a recent study by Boostani et al. (2017) attempted to circumvent this limitation by applying the same PSG labeled datasets (normal and patient groups) to five classification algorithms (GMM Classifier, LDA + NC Classifier, KNN Classifier, LDA + NC Classifier, Random Forest Classifier) developed by five different research groups [85]. It was found that the seven feature, Random Forest Classifier had the best classification accuracy (87.06% and 69.05%, for normal and patient datasets, respectively). The researchers then applied each classifier to the feature sets utilized within each of the aforementioned models and found again, that the Random Forest Classifier consistently had the highest performance accuracy.

Other studies have attempted to assess the accuracy of automated sleep stage detection in wearable devices against the gold-standard PSG, with varying levels of success. Namely, Fonseca et al. (2017) analyzed PSG vs. actigraphy and PSG vs. PPG with actigraphy data using a Bayesian LDA classifier to find that accuracy increased with the addition of a PPG signal to the model [70]. Similarly, Walch et al. (2019) applied raw acceleration and HR data to a logistic regression, KNN, random forest, and a neural net classifier to determine sleep vs. wake and subsequent sleep staging against PSG, reporting the best performance from the neural net classifier with an accuracy of 91.3% and 65.2% for sleep vs. wake and sleep staging, respectively [79]. SVM and XGBoost classifiers have been reported to provide similar degrees of epoch by epoch accuracy of 73.1% [88]. As demonstrated, data signals can be analyzed via numerous different classifiers, each found to have varying degrees of accuracy for the assessed context within healthy populations.

Once the model is trained and cross-validated on a sufficient sample of data and has achieved an acceptable level of accuracy, it can be used on a novel dataset. Performance of a model is traditionally gauged on a model’s ability to classify a novel dataset and expressed in terms of accuracy, sensitivity, specificity, or Cohen’s kappa. Accuracy can be reported in a variety of fashions but most often aims to describe the percentage of epochs that were correctly labeled by the algorithm as compared to PSG. However, there is a significant drawback in percentage agreement—it does not consider the level of agreement that is expressed between two observations that are unrelated (the ‘by chance’ effect) [87]. The Cohen’s κ aims to address this effect by providing a chance adjusted measure of agreement ranging between 0 and 1 to compare the inter-rater agreement of a PSG scorer with the automated sleep classifier. A value closer to 1 suggests near perfect agreement, whereas a value closer to 0 indicates that the model’s ability to predict a specific sleep stage is no better than chance itself [73].

The physiologic response of individuals during sleep is highly variable; algorithms based off an individual’s normative values rather than a population would result in a more accurate classifier [90]. However, the feasibility associated with developing a novel algorithm specific to each individual is unrealistic, as it requires a significant amount of previously pre-labeled PSG data and significant computational resources. Therefore, the widely utilized approach for sleep classification is to implement user adaptation methods in the form of unsupervised clustering. Clustering algorithms associate physiologic signals from the new subject with a pre-trained, subject group that best matches the individual. Other algorithm architectures may utilize a combination of group features and individual features to drive sleep classification [24].

Success in autonomous sleep staging is largely dependent on the quality of the data used to train the model. However, once optimized and validated, automated sleep classification algorithms have the potential for numerous advantages, including the ability to monitor sleep in a free-living environment that may more accurately depict an individual’s normative sleep patterns. Validation of automated sleep classification algorithms are largely limited by the lack of independent validations on the direct sensor outputs and post-processed data outputted by the proprietary algorithms developed by manufacturers. This is further limited by the fact that devices often undergo firmware and software updates by the manufacturer; therefore, even if a device has been previously validated against PSG, it will require extensive future validations, with prior validations deemed irrelevant [79].

## 7. Success in Estimating Sleep

At large, this intricate process presents many opportunities for error. A significant contribution of device accuracy is in the success of algorithms; however, while the recent transitions toward multi-modal devices presents greater opportunities for the field of wearable devices, failure to produce high quality sensors that support the intended conditions will restrict the ability to improve device accuracy. It is also important to note that many commercial devices that claim to measure sleep, are primarily advertised as “activity trackers” with many purported abilities other than sleep monitoring. Though all advertised claims should be treated similarly, end-users should be aware that proclamations of an accurate, cost-effective, all-encompassing solution for health monitoring is largely unrealistic.

Despite the multitude of validations in this field, few studies have attempted to consider a justification for the broad range of values determined in validation studies. That is, given the obvious capabilities of devices, how do they perform relative to other devices? Given the relative capacities of wearable devices, this justification for varying degrees of device performance can be supported by third-party validation efforts. Table 2 summarizes the general advertising claims of many popular commercial devices; in combination with sensor specifications for each device listed previously in Table 1, this information demonstrates the relationship between device engineering and purported claims as they relate to device accuracy.

While the field has spent nearly a decade attempting to keep validation efforts up to speed with the rapidly developing device models and software versions, comparability of third-party validation efforts across devices is ultimately limited due to the varying conditions and methodologies utilized therein. However, few studies have attempted to measure the relative device accuracy of multiple devices concurrently. Stone et al. (2020) assessed the overall nightly reports of various sleep devices against an EEG device, including the Fatigue Science Readiband, Fitbit Ionic, Garmin Vivosmart 4, OURA, and Polar A370 [91]. Unsurprisingly, the two most accurate devices in identifying states of sleep versus wake were the OURA and the Fitbit Ionic, which were the only two devices tested therein that were equipped with accelerometers and PPG sensors utilizing red or infrared light sources. Similarly, Chinoy et al. (2020) also found the device with red and infrared (IR) PPG to have the highest accuracy of the five wearables tested against PSG, and found that devices only equipped with an accelerometer were able to more accurately classify sleep and wake over devices equipped with green LED PPG sensors [92]. These findings support the notable contribution the sensor specifications can have in overall device accuracy. During sleep, when there is relatively little body movement, red and infrared light sources provide the greatest opportunity for accurate HR and HRV measures.

Though many of the devices listed in Table 1 and Table 2 have yet to have third-party validations published against PSG, it can be well demonstrated between the sensor information in Table 1 and the purported measures and advertised claims in Table 2, why devices advertise the opportunity to quantify sleep. For example, the Fatigue Science Readiband, which is only equipped with an accelerometer, only claims to differentiate sleep and wake, likely because it lacks the capacity to measure HR and related metrics, which would make sleep staging attempts nearly impossible. Other devices discussed all have a means of measuring HR and related measures, whether it be via PPG or ECG. Additionally, it is important to note that while many of these devices claim to measure sleep, they are not all marketed primarily as sleep assessment devices. Some devices that are primarily advertised for exercise or sport applications, are more likely to be equipped with PPG sensors utilizing a green LED light source to improve measurability during high movement activities. These diverging aims often contribute to overall device accuracy in sleep quantification, as sensors are engineered for a “best fit” for most purported capabilities.

## 8. Remaining Challenges for Accurate Sleep Monitoring

The primary problem that exists surrounding sleep wearable accuracy is the gap between methodology and the required information needed to draw informed conclusions. Considerable overlap exists in PSG scoring of primary physiological signals that are indicative of a given stage due to the complexity of the data; wearable devices, typically positioned peripherally, then collect fewer data types and collect signals that are different in nature, yet classified the same. This lack of information also restricts the ability to account for non-normative physiology (i.e., effects of vascular stiffness on BP or PPW) or confounding factors that exist in a free-living environment (i.e., effects of nicotine or caffeine on cardiorespiratory patterns).

The use of these devices in a free-living environment must also be considered when determining device accuracy. While models are trained to identify various patterns as artifact, one must expect that user interaction with the environment continues independent of consciousness. That is, confounding factors can contribute to measurement or algorithmic error, as well as alter normal physiology; for example, when considering sleep patterns, the occurrence of movement from bed partners will cause accelerometers to pick up on movement of the user, not produced by the user, resulting in incorrect classification based on this bio-signal alone.

Beyond accurate data collection, cleaning, and calculation, the final automated classification of state or stage is of great difficulty. Since most companies choose not to disclose information surrounding their proprietary algorithms, a great deal of variation exists from company to company regarding expected physiological parameters. Not only do training paradigms and populations vary, but no current standard exists for sleep stage classification using solely physiological parameters obtained with the wearable methods discussed in this review; the lack of standards for signals discussed herein, such as those described by the AASM for sleep staging with PSG [16], further exacerbates the variation in the field of wearable sleep trackers. It is important to note that numerous studies have been conducted to identify the accuracy of PSG data when staged based upon cardiorespiratory factors alone, which are the primary data signals utilized for sleep staging in wearable devices; these studies demonstrate similar degrees of staging accuracy as many wearable devices as reported in validation studies [78,93]. Further, few studies consider the differences in methodology when conducting validations, failing to contribute the resultant uncertainty to specific contributing factors; for instance, an algorithm aimed to classify sleep based upon cardiac features was found to have significantly worse performance when applied to PPG features as compared to ECG features, despite similar accuracies in HRV parameter estimation [94]. Additional studies such as these, aimed toward isolating performance variations, are needed in the field and are vital for the improvement of overall device accuracy.

With even the most accurate data collected from equipped sensors, wearable devices still require sleep to elicit black-and-white behavior during homologous stages, which could not be further from reality. The highest rates of error reported by validation studies likely result from insufficient contextual information necessary for accurate classification. Across wearable devices, a notable issue persists in differentiating light sleep from wake when movement produced by an individual remains static; this predicament is well demonstrated by consistent overestimations of sleep durations across third-party validation efforts [68]. Among stages, REM, aptly named paradoxical sleep, presents the highest degree of difficulty for classification by result of physiological variation of normal parameters; phasic REM closely resembles the physiology of wake, whereas physiology during tonic REM can easily be mistaken as light sleep [10]. Discernment in that sleep physiology is fluid and variable remains a consequential barrier for accurate free-living sleep tracking.

Despite the limitations, the opportunities presented by wearable devices for free-living sleep evaluation propose a solution for data collection that is otherwise currently unobtainable. These wearables should not be seen as an alternative to gold standard methodologies, but rather a potential solution for challenges left unsolved from PSG: unobtrusive monitoring of natural behavior, with the ability to assess longitudinally. Additional considerations should be made by the end-user, whether it be consumers, researchers, or clinicians, based upon prospective outcomes.

### 8.1. Utility and Challenges for Consumer Use

Initially, the target population that fueled the rise in popularity of these “at-home sleep trackers” was the general consumer who strived to garner a greater understanding of their health. In spite of this intent, the opportunity to target the uninformed was quickly acknowledged by marketing departments; this challenge remains throughout the market for companies who aim to educate customers, but also fail to disclose the confidence expressed in the information they provide.

A recent shift has occurred for many devices in the field in terms of the user interface they provide. The simple reports on sleep duration that were initially typical have transitioned towards a higher number of data values, and more notably, interpreted data. In expansion of the ranges of data types reported, some devices like OURA have begun incorporating end-user educational information into their user interface. Application of information in this way is an opportunity for a consumer to easily acquire knowledge surrounding the science behind their health while providing both context and impact.

Though convenient, this interpretation of the data, in combination with overall data reporting without reference to accuracy rates, can be inherently dangerous for the naïve consumer. Companies too often fail to consider the psychology of the consumer and how they perceive device-obtained data. While no company will admit to the potential for poor device accuracy, failure to disclose the low confidence behind reported data can be a safety risk for the user; as such, class-action lawsuits have succeeded based upon inaccuracies, especially given the injury risk that can stem from altered perception and overestimation of personal capabilities after being provided with inaccurate data from a wearable device [95].

### 8.2. Utility and Challenges for Research

The use of wearable sleep trackers for research surrounding free-living patterns is becoming increasingly popular as devices and technology develop. Of greater concern for accuracy as compared to the general consumer, it must be acknowledged that the use of these devices will only be appropriate in certain scenarios. While keeping in mind any validation efforts made regarding a device, multi-modal devices are the first opportunity that the research community has had to obtain free-living sleep physiology in large populations.

In beginning to bridge the limitations that still existed with the use of actigraphy, many consumer wearables still limit considerable utilization by the research community. In contrast to actigraphy data, which is traditionally only presented in raw form to practitioners who purchase the software, few multi-modal wearable devices have provided third-party access to raw forms of device-collected data. In addition to the rare disclosure of proprietary algorithms, this opportunity for error in combination with the minimal applications for summative data values, leaves many researchers skeptical. Both raw data access and transparency of algorithms should continue to be requested of manufacturers as these markets continue to develop.

Considering the limitations, efficacy for the use of commercial sleep wearables in research is ultimately determined by the research question. Many devices have been found to estimate sleep durations and sleep efficiency with relatively little error or requirements from the end-user [91,92]. Similar findings exist surrounding physiology in terms of HR, HRV, and RR [96,97]. However, few researchers will be comfortable utilizing sleep staging classifications reported from these devices, as most devices consistently exhibit error rates over 30% [91,98]. While the question of ‘how accurate is accurate enough?’ will never be answered simply, this question does pose significant context for whether commercial multi-modal devices may be appropriate for use in research [99].

### 8.3. Utility and Challenges for Clinical Applications

The use of commercial sleep wearables in monitoring patients for clinical use is restricted by many of the same limitations presented for research use. Though strongly impacted by device accuracy and restricted access to raw data, information surrounding model training for data analytics presents further issues for use in some populations. Notably, populations with non-normative sleep physiology, such as patients with sleep disorders, are particularly susceptible to high error rates [100,101]. Algorithms are trained to detect physiological signals that are characteristic of a specific state under healthy conditions, but when physiology changes independent of sleep, false classifications are likely.

Advancements in the use of wearables in a clinical setting have considered the opportunity presented for health disorder screening and diagnostic purposes. As mentioned, most consumer wearables are trained on populations that are deemed to exhibit healthy and normative nocturnal physiology; in addition to the potential camouflaging this can demonstrate on undiagnosed disordered sleep, this limits the accuracy of many models for disordered populations. For example, sensors and cleaning algorithms aimed toward sleep monitoring are trained in a population undergoing minimal movement during the time of measurement; in contrast, if used for screening a patient with REM behavior disorder (RBD), traditional classifications will not be reflected in signals obtained by this patient. Other clinical uses, such as monitoring patients with sleep apnea, may also yield incorrect stage classifications; while clear differences exist in the respiratory patterns of sleep apnea patients, significant differences in HRV parameters have also been identified, both of which are key contributors for sleep staging in wearables [102].

The development of screening and diagnostic tools for use of wearable sleep devices in clinical setting would be of great value, and could provide benefit for physicians in both diagnosis and the development of treatment plans. Though, the challenge remains that algorithms utilized by these devices must not be dependent on basic physiological signals for staging; rather, developments may focus on the deviations from traditional physiology, which could provide insight suggestive of more specific conditions that could be further evaluated by a sleep study. Physiological monitoring could also provide benefit for monitoring the efficacy of treatments, and would place fewer limitations on model training for specific populations, which may vary in accuracy throughout the treatment process. While consideration of the clinical aim and the question of ‘how accurate is accurate enough?’ still remains highly relevant for practitioners, it is important that clinical decisions not be solely based off exploratory methodologies. Using commercial devices in a way in which they were not designed to operate can lead to missed or incorrect diagnoses with severe consequences for the patient.

## 9. Conclusions

With consideration to the complexities of traditional polysomnography, as well as the variation that still exists with its interpretation, it could be argued that wearable devices have overcome considerable challenges regarding free-living sleep monitoring. Given that algorithms are often trained using polysomnographic scoring that can vary by 20–30% between sleep technologists [18,19,20,21], it is nearly impossible for the accuracy of secondary signal collection and automatic interpretation to near perfection. The limitations of device assessment against the gold standard will persist, much beyond the control of the end-user, given that the two methods aim to evaluate different physiological signals entirely. However, it can be well understood by the end-user what variables can contribute to device accuracy, including those effecting the collection and interpretation of these distal signals. Variations in sensor types and their specifications will contribute to the accuracy of captured secondary signals in a given manner. Devices with components designed to prioritize the capture of sleep related phenomena are likely to result in greater accuracy, as compared to devices that attempt to measure a broad range of physiological behaviors, such as during both exercise and sleep. In understanding the capabilities of wearable sensors in measuring sleep related physiology, the realm of use for commercially available devices should be relative to the individual objective set by the end-user. Wearable devices should not be considered an alternative to the gold standard polysomnography, but can provide meaningful insight surrounding naturalistic behavior when maintaining vigilance in regards to its application.

## Figures and Tables

**Table 1:** This table includes device specifications for common commercially available wearable technologies that claim to assess sleep. Information regarding each device has been obtained via its respective company resources, where available for release, including specifications of the photoplethysmographic sensor, if applicable, for the device. It is important to note that while a sensor may be embedded in a device, it does not ascertain that it is used in sleep evaluation; sensors denoted by an asterisk (*) have been confirmed by company resources to not participate in sleep quantification efforts. Reflective arrangements of PPG sensor components listed as reflective are direct unless otherwise specified. (ECG, electrocardiography; gen., generation; LED, light emitting diode; N/A, not applicable; NTC, negative temperature coefficient; PPG, photoplethysmography).

Device	Location	Sensors	PPG Specifications		
			Type	Light Source(s)	Arrangement
Amazon Halo	Wrist	Accelerometer	Contact	Green LED	Reflective
		PPG			
		Temperature Sensor			
Apple Watch Series 6	Wrist	Accelerometer	Contact	Infrared Green LED Red LED	Reflective
		ECG			
		Gyroscope			
		PPG			
Fatigue Science Readiband	Wrist	Accelerometer (3-axis)	N/A	N/A	N/A
Fitbit Ionic	Wrist	Accelerometer (3-axis)	Contact	Infrared Red LED	Reflective
		PPG			
Fitbit Sense	Wrist	Accelerometer (3-axis)	Contact	Infrared Green LED Red LED	Reflective
		ECG			
		Gyroscope			
		PPG			
		Temperature Sensor			
Garmin Vivosmart 4	Wrist	Accelerometer	Contact	Green LED	Reflective
		PPG			
OURA (2nd gen.)	Finger	Accelerometer (3-axis)	Contact	Infrared	Reflective (angled)
		Gyroscope *			
		PPG			
		Temperature Sensor (NTC)			
Polar A370	Wrist	Accelerometer	Contact	Green LED	Reflective
		PPG			
Polar Grit X	Wrist	Accelerometer (3-axis)	Contact	Green LED Red LED Yellow LED	Reflective
		ECG/Electrical conductance			
		PPG			
Whoop 3.0	Wrist	Accelerometer (3-axis)	Contact	Green LED	Reflective
		Capacitive Touch Sensor *			
		Gyroscope (3-axis)			
		PPG			
		Temperature Sensor *			

**Table 2:** Advertised claims and specifications of various popular devices on the market. Two asterisks (**) denotes availability with an additional paid monthly subscription. (d, days; gen, generation; hrs, hours; m, month; RR, respiration rate; Temp, temperature).

Device	Location	Marketed Terminology	Advertised Capabilities				Battery Life	Cost	Year of Release
			Sleep/Wake	Sleep Staging	Nocturnal Physio	Other			
Amazon Halo	Wrist	“Health & wellness band”	X	X		Temp	7 d	$100 + $4/m	2020
Apple Watch Series 6	Wrist	“Watch”	X				18 hrs	$400	2020
Fatigue Science Readiband	Wrist	“Sleep collection device”	X				30 d	$500	2010
Fitbit Ionic	Wrist	“Smartwatch”	X	X	X		5 d	$250	2017
Fitbit Sense	Wrist	“Smartwatch”	X	X	X **	Temp	6 d	$300	2020
Garmin Vivosmart 4	Wrist	“Sport and fitness tracker”	X	X			7 d	$130	2018
OURA (2nd gen.)	Finger	“Sleep tracking smart ring”	X	X	X	Temp, RR	7 d	$300	2018
Polar A370	Wrist	“Fitness tracker watch”	X				5 d	$150	2017
Polar Grit X	Wrist	“Outdoor multisport watch”	X	X	X	RR	7 d	$430	2020
Whoop 3.0	Wrist	“Recovery, fitness, and sleep tracker”	X	X		RR	5 d	$30/m	2019

## Data Availability

Not Applicable.
